# Whether New Cooperative Medical Schemes Reduce the Economic Burden of Chronic Disease in Rural China

**DOI:** 10.1371/journal.pone.0053062

**Published:** 2013-01-09

**Authors:** Shanshan Jing, Aitian Yin, Lizheng Shi, Jinan Liu

**Affiliations:** 1 Center for Health Management and Policy, Shandong University, Ji’nan, Shandong Province, China; 2 Department of Global Health Systems and Development, School of Public Health and Tropical Medicine, Tulane University, New Orleans, Louisiana, United States of America; Tehran University of Medical Sciences, Islamic Republic of Iran

## Abstract

**Background:**

The New Cooperative Medical Scheme (NCMS) provides health insurance coverage for rural populations in China. This study aimed to evaluate changes in household catastrophic health expenditure (CHE) due to chronic disease before and after the reimbursement policies for services of chronic disease were implemented to provide additional financial support.

**Methods:**

The study used data from the household surveys conducted in Shandong Province and Ningxia Hui Autonomous Region in 2006 and 2008. The study sample in village-level units was divided into two groups: 36 villages which implemented the NCMS reimbursement policies for chronic diseases as the intervention group, and 72 villages which did not as the control group. Health care expenditure of more than 40% of household’s non-food expenditure was defined as a household with CHE (i.e., impoverishment). The conceptual framework was established based on the Andersen socio-behavioral model of health care utilization to explore how the NCMS reimbursement policies impacted health expenditures. A difference-in-difference model was employed to compare the change in the proportion of households incurring CHE due to chronic disease between the two groups over time.

**Results:**

The households that participated in the NCMS were less likely to become impoverished (P<0.05). In addition, the households with both male household head and higher income level were protective factors to prevent CHE (P<0.05). Young households with preschool children suffered less from CHE (P<0.05). The effect of the NCMS reimbursement policies for chronic disease on the CHE was negative, yet not statistically significant (p = 0.814).

**Conclusions:**

The NCMS coverage showed financial protection for households with chronic disease. However, the NCMS reimbursement policies should be strengthened in the future.

## Introduction

Chronic disease accounts for an estimated 80% of total deaths and 70% of total loss of disability-adjusted-life-years (DALYs) in China [1], and the estimated losses in national income from heart disease, stroke and diabetes are US$18 billion in China in 2005 [2]. The Fourth Chinese National Health Services Survey in 2008 showed that 270 million Chinese people were diagnosed with at least one chronic disease, and about 82% of deaths and 70% of DALYs lost were caused by chronic diseases. By 2020, the number of deaths attributable to chronic diseases was predicted to raise to 85% of national mortality statistics [3].

Chronic diseases pose a heavy financial burden for rural residents. Average inpatient expenditure on common types of chronic disease, such as hypertension and diabetes, can cost 1.5 times the annual per capita income of rural residents [4]. It is estimated that 54% of total household medical expenditure on chronic disease is spent on outpatient services. The statistics are ever higher for hypertension (67%) and for rheumatoid arthritis (74%) [5]. One study in Shandong and Gansu provinces estimated the annual per capita outpatient expenditure on chronic disease at half of total annual medical expenditure [6].

The burden of health care costs on household financial situation depends on the country’s health care system and the ability of individual households to pay [7].In a recent global review of household catastrophic health payments, the role of health insurance was emphasized as a key instrument in reducing the family burden [8]. The high costs of health care may not be family burden under full coverage health care systems, while even a low level of out-of-pocket cost without sufficient coverage can be catastrophic for poor households [9]. To secure access to universal health care at an affordable price, it will be necessary to increase the extent of reimbursement and reduce reliance on out-of-pocket payment [10], [11].

China had developed a successful community-based health insurance system called the Cooperative Medical Scheme (CMS) in rural areas since the 1950s. By the late 1970s, approximately 90% of the rural population was covered by the CMS. The shift to a market economy and the demise of the rural communes in the 1980s led to the collapse of CMS [12]. This left large sectors of the rural population without health insurance coverage in the 1990s [13]. The Chinese government put forward the New Cooperative Medical Scheme (NCMS) for rural residents in 2003. This scheme involves voluntary participation, heavy subsidization, and a design to reduce the financial burden of illness on the rural population. The Ministry of Health takes the overall responsibility to manage and supervise the scheme while the responsibilities for policy implementation are decentralized to county-level governments. One of the NCMS’s key objectives is to effectively reduce rural people’s economic burden of health services and to relieve impoverishment through protection against catastrophic health expenditure.

Given limited funding and the goal of protecting households from medical impoverishment, the NCMS programs in the majority of counties focused on providing insurance against “catastrophic” inpatient expenses. However, there is increasing empirical evidence that ambulatory services and drugs, rather than hospitalization, are the primary contributors to medical impoverishment [14], [15], [16]. Patients with chronic conditions make frequent visits and incur high medication costs that are not reimbursed under the NCMS catastrophic coverage model. The Yip and Hisao et al study [16] found that 11.6% of the rural poor households became impoverished due to outpatient expenses associated with chronic disease. The need to control the escalating health expenditure, a large part of which is attributable to chronic disease, has been an increasing concern on improving the NCMS programs in China.

Recently, the NCMS programs of some counties have implemented some initiatives of reimbursement policies specifically for patients with chronic disease. The policies vary from county to county on the reimbursement scope and level, largely depending on their funding pooling and the local incidence of chronic disease. These reimbursement policies of the NCMS programs have some common characteristics: (a) reimbursement of outpatient expenditure; (b) waiver of the outpatient reimbursement deductible and increase in the ceiling level; (c) partial coverage of the common and serious types of chronic disease; (d) the covered chronic disease identified by the experts in the designated health institutions and reimbursement only for the expenditures in these health institutions; and (e) the higher reimbursement rate of the covered chronic disease (40% on average) than other diseases (25%). In the sample area, Changle County and Dong’e County in Shandong Province implemented the NCMS reimbursement policies for outpatient expenditure of chronic disease in July 2007 and July 2008, respectively. The details of the policies in the two counties are presented as following:

### Changle County

Patients with hypertension or diabetes who are enrolled in the standardized chronic disease management program are reimbursed by 80% of their essential drug expenditure at village clinics and township hospitals. The essential drugs for hypertension include nifedipine, atenolol, hydrochlorothiazide and enteric-coated aspirin tablets. The essential drugs for diabetes include glibenclamide and metformin hydrochloride. The reimbursement rate of other expenditures for chronic disease is at the rate of 25%.

### Dong’e County

A Chronic Disease Fund was established and subsidized 14 types of chronic disease including hypertension, diabetes, tuberculosis, anemia, etc. Patients were reimbursed 40% of the outpatient expenditure associated with the listed chronic disease at village clinics and township hospitals. The reimbursement rate is 15% higher than that of other diseases.

The objective of this paper is to examine the impact of the NCMS reimbursement policies for chronic disease on the household CHE. It is important evidence for policy makers to improve the NCMS programs to provide financial protection to patients with chronic disease.

## Methods

### Data and Sampling

The data are derived from the household surveys conducted in Shandong Province and Ningxia Hui Autonomous Region in May 2006 and August/September 2008 [17], [18], [19]. It is part of a European Union-funded project aiming to improve the design of rural health insurance in rural China and Vietnam entitled ‘Bringing health care to the vulnerable developing equitable and sustainable rural health insurance in China and Vietnam (RHINCAV)’ [20], [21].Using the structured questionnaires in the face-to-face interviews, individuals with one or more chronic conditions reported basic social and economic characteristics (income and expenditure levels and NCMS membership); health care utilization, costs, and levels of NCMS reimbursement where applicable. In the questionnaire, the items of total expenditures for health care and the expenditure due to chronic diseases was separated, when the interviewees were asked to report the name or main symptoms of each reported chronic disease, the level of health care providers who diagnosed them and the expenditures of each visit was also reported. The total expenditures include outpatient, inpatient and drug store expenditures. Chronic disease was defined as an illness or symptom with at least 3 months continuous or intermittent presentation within the last 12 months. The rural residents receive annual physical checkup covered by the NCMS. Therefore, their health information is regularly collected and the patients with chronic conditions can be identified.

The multistage sampling was used, the selection of the two provinces aimed to represent different levels of socio-economic development and geographic areas in China. Three counties were selected from Shandong (Zhangqiu, Changle, and Dong’e) and three from Ningxia (Zhongning, Yongning and Qingtongxia). The selection of counties was based on the following criteria: 1) all the counties have an existing NCMS program; 2) the county governments are willing and able to collaborate with the research project; and 3) the three counties should have different levels of socio-economic development and be located in a similar geographic area. Three townships in each county and six villages in each township were selected using similar criteria to the selection of counties. In each village, 60 households in 2006 and 30 households in 2008 was randomly sampled.2998 households in 2006 and 1681 households in 2008 responded that one or more members in the household had suffered from a (self-identified) chronic disease. This paper presents an analysis of the households with chronically ill patients and the data was aggregated to village-level analytical units with a total of 108 villages in the two wave surveys. Changle and Dong’e were in the intervention group, including 36 villages; the other 4 counties were in the control group, including 72 villages.

### Statistical Model

The hypothesis of this study is that after implementing the reimbursement policies, there are fewer households with catastrophic health expenditure due to chronic disease. CHE was defined as health expenditure 40% greater than the ability of the household to pay, as described by Xu et al. [8]. Ability to pay was defined as the household non-food expenditure in this study.

To measure the impact of the NCMS reimbursement policies for outpatient expenditure on the likelihood of incurring CHE for those households, this study employed a difference-in-difference (DID) model between the intervention group and the control group. The economic burden was measured by a proportion of households incurring CHE due to chronic disease. The difference-in-difference model is of the following form:
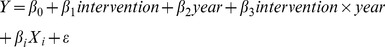



In this equation, the dependent variable was the proportion of households which had catastrophic health expenditure due to chronic disease. *Intervention* was a dummy variable of 1 for intervention group and 0 for control group. *Year* is a dummy variable of 0 for before the NCMS reimbursement policies for chronic disease (wave 1: year 2006) and 1 for after policies (wave 2: year 2008).*β_3_* is the coefficient of the difference-in-difference estimate, which measures the impact of the policies on reducing the CHE between the two groups. X_i_ is a vector of the household characteristics, including economic status, the gender of household head, and the occupational and educational situation of the household head. The NCMS participation rate was also controlled for in the model.

## Results

In the baseline survey, the total number of households was 989 (32.99%) in the intervention group and 2009 (67.01%) in the control group. In the second wave survey, the total number of households was 526 (31.29%) in the intervention group and 1155 (68.71%) in the control group. Table 1 presents the household characteristics of the two groups.

**Table 1:** Household characteristic of the two groups.

household characters	2006		2008	
	Intervention group (%)	Control group (%)	Intervention group (%)	Control group (%)
Total households	989 (32.99)	2009 (67.01)	526 (31.29)	1155 (68.71)
Income quintiles				
1(poorest)	209 (21.13)	392 (19.51)	119 (22.62)	221 (19.13)
2	196 (19.82)	403 (20.06)	126 (23.95)	212 (18.35)
3	196 (19.82)	404 (20.11)	105 (19.96)	232 (20.09)
4	212 (21.44)	388 (19.31)	104 (19.77)	233 (20.17)
5(richest)	176 (17.80)	422 (21.01)	72 (13.69)	257 (22.25)
Gender of HH head				
male	914 (92.42)	1823 (90.74)	492 (93.54)	1061 (91.86)
female	75 (7.58)	186 (9.26)	34 (6.46)	94 (8.14)
Insurance of HH head a ***				
NCMS	924 (93.43)	1779 (88.55)	517 (98.29)	1092 (94.55)
Employee insurance	8 (0.81)	17 (0.85)	6 (1.14)	10 (0.87)
Commercial insurance	25 (2.53)	184 (9.16)	16 (3.04)	150 (12.99)
No insurance	39 (3.94)	188 (9.36)	3 (0.57)	41(3.55)
Occupation of HH head***				
famer	789 (79.78)	1433 (71.33)	423 (80.42)	934 (80.87)
employee	139 (14.05)	495 (24.64)	82 (15.59)	190 (16.45)
civil service	61 (6.17)	81 (4.03)	21 (3.99)	31 (2.68)
Education of HH head**				
primary and below	460 (46.51)	1045 (52.02)	198 (37.64)	522 (45.19)
junior high school	420 (42.47)	780 (38.83)	240 (45.63)	479 (41.47)
senior high school	95 (9.61)	162 (8.06)	79 (15.02)	130 (11.26)
College and above	14 (1.42)	22 (1.10)	9(1.71)	24 (2.08)
65+ years of age**	336 (33.97)	605 (30.11)	172 (32.70)	304 (26.32)
preschool children***	152 (15.37)	490 (24.39)	113 (21.48)	299 (25.89)

a The household head (HH) may participate in more than one insurance system.

** Significance at 5%.

*** Significance at 1%.

Compare the intervention group and control group in baseline survey.

### The Household Socio-demographic Characteristics in Baseline Survey

In the baseline survey, a total of 2998 households was selected and divided into 5 groups according to the household annual gross income. The economic status was comparable between the intervention group and control group (p = 0.212). The gender of household heads was mainly male (92.42% in the intervention group and 90.74% in the control group, p = 0.126). The NCMS participation of household head was higher in the intervention group (93.43%) than in the control group (88.55%) and was statistically significant (p<0.001). Most of the household heads were farmers in the intervention group (79.78%) and the control group (71.33%, p<0.001). Compared with the control group, the intervention group has higher educational status of the household heads (p<0.05). The intervention group had more proportion of households with people of age 65 years or older, than the control group (33.97% vs. 30.01%, p<0.05). The intervention group had fewer preschool children than the control group.

### The Difference-in-difference Changes of Household CHE

Table 2 shows the proportion of household CHE in year 2006 and 2008 between intervention group and control group. Two groups did not differ much in the household CHE reduction before the policy was implemented (6.19% vs. 7.21%). They differ more after the policy was implemented (3.56% vs. 5.11%). In other words, two groups produced a decrease in the percentage of household CHE (intervention group: 2.64% vs. control group: 2.10%). However, the DID estimate was negative (−0.53%). The co-payment of the intervention group also reduced in the study, from the average number of 1021.66 RMB in 2006 to 701.69 RMB in 2008.

**Table 2:** Average proportion of CHE before and after NCMS reimbursement policies.

group	Before mean (S.D.)	After mean (S.D.)	Diff mean (S.D.)
intervention	6.19(3.61)	3.56(2.43)	−2.64(4.73)
control	7.21(4.19)	5.11(4.68)	−2.10(5.75)
Diff mean (S.D).	−1.02	−1.55	−0.53(DID estimate)

### The Impact of the Policies on Household CHE

Table 3 shows the difference-in-difference regression results. Controlling for household demographic characteristics, the coefficient of the interaction term was negative but not statistically significant, suggesting that the reimbursement policies for chronic disease in the NCMS programs were not significantly effective in reducing household CHE. Household per capita gross income was found to significantly relate with household CHE (P<0.05). The study also found that households with female household head were more likely to drop into impoverishment due to chronic disease (p<0.05). The results also showed that the household whose head participated in NCMS could protect the families from catastrophe caused by chronic diseases (p<0.05). It is a fact that in rural China the family members are generally required to enroll together in NCMS. Therefore, the NCMS significantly reduced the household CHE. Another factor that mitigated the household CHE was the households with preschool children (p<0.05).

**Table 3:** The difference-in-difference regression model of household CHE (n = 108).

Variables	Description	coefficient	p-value
D-I-D			
Year	dummy variable being 0 for 2006,1 for 2008	−.011	.105
Group	dummy variable being 0 for control group,1 for intervention group	−.014	.098
Group×year	interaction variable	−.003	.814
Control variables			
Ln income	gross income per capita	−.017	.013
Male HH head	proportion of male HH head	−.092	.015
Education	proportion of HH head have elementary or lower education	−.005	.810
NCMS	proportion of HH head participate in NCMS	−.089	.007
Occupation	proportion of farmer HH head	.024	.254
65+	proportion of HH which have 65+ members	.042	.075
Preschool children	proportion of HH which have preschool children	−.052	.020

R^2^ = 0.199; F = 5.089; p = 0.000.

## Discussion

In this study, the NCMS reimbursement policies for chronic disease only found numerical improvement in preventing households CHE. These policies of the two counties were both in the initial stage of implementation when the household survey was conducted. With either the low reimbursement rate or narrow coverage, the insignificant effect of the reimbursement policies seemed to be reasonable.

Future policies should be strengthened both in margins of intensity (higher reimbursement rate) and extensity (e.g., other chronic disease). The NCMS in Changle County has already expanded the scope of the reimbursement of chronic diseases since 2009, adding cerebrovascular disease, chronic aplastic anemia, cancer chemotherapy and uremia into the coverage list. Furthermore, the government’s subsidy to the NCMS has been improving; the premium level has risen to 250 Yuan (39 US$) in 2011. With the continuous improvement in the NCMS funding level, the impact of NCMS reimbursement policy on chronic disease patients needs re-evaluation.

The NCMS participation was found to be a protective factor to reduce the household CHE, consistent with other studies [19], [22]. A previous study that estimated the impacts of NCMS in 2003 and 2005 demonstrated that NCMS programs have reduced the incidence of catastrophic spending in the poorest group [23]. It is still not conclusive in this study that the NCMS has played an effective role in reducing the economic disease burden of rural people. Furthermore, until 2008 the participation rate of NCMS in the six study counties had exceeded 95% on average, and the average premium had risen from 25 RMB in 2005 to 85 RMB in 2008. With the expansion of coverage and rising funding level, the ability of the NCMS programs on reducing the economic burden of chronic diseases has been enhanced. Meanwhile, we should also be aware that the NCMS programs still focus their reimbursement coverage on inpatient expenditure, and patients with chronic illness are more likely to get effective reimbursement when they incur an acute incidence or use inpatient services.

Some other factors that impact household CHE were found in this study. The household income is inversely associated with poverty caused by health expenditure. Extra attention should be paid to the poor population because the previous study found that NCMS programs did not improve out-of-pocket spending among the poorest decile [23]. In China, the Medical Aid System can play a key role in this circumstance to give extra financial assistance for those who are the poorest people, including the rural poorest households with chronic disease.

The study has also found that the households with female head were more likely to be negatively affected by the financial burden of chronic disease. This can be explained by the fact that the female head of a household always has unfavorable conditions to provide better economic status for a family, such as the main labor force of the family, and the provider of the main source of income for the family.

Furthermore, the observation that households of preschool children are less likely to suffer from catastrophes can be explained by the fact that these households with young children are more likely to have younger and healthier parents [24]. Age could be a confounding factor behind this as another explanation. Although the factor that households have elderly people was not statistically significant to household CHE in this study (p = 0.075), results indicated that having elderly people in a family may also increase the possibility of household CHE. Similar results were found in a study in Turkey [24]. Further effort is needed to reduce the financial burden for certain disadvantaged groups, such as households that have elderly people.

This study has several limitations. First, this study is not a controlled trial but rather a “natural experiment”, the main characteristics in the two groups couldn’t be controlled very well, thus the treatment group was not totally comparable to the control group at baseline. Two selected provinces had many differences and integrated village-level data may not reveal a lot of differences. The intervention and control counties satisfy the parallel trend (pre intervention) should be further examined to improve the DID model specification. Second, the estimations were based on self-reported chronic disease, rather than on those diagnosed by a physician. Chronic illness and its costs may be either over- or under-reported, due to the lack of a standard set of criteria used to distinguish chronic from acute illness. However, the questions used to elicit self-reporting of chronic disease were drawn from the National Health Survey, which has been validated nationally. In addition, if we had only included chronic diseases diagnosed by a physician this could underestimate the disease burden, considering the barriers to obtaining a formal diagnosis. Third, we did not include data on the indirect costs of seeking care, such as those associated with transportation, food, and accommodation, which are often substantial in rural China or loss of earnings associated with time for medical care. Our estimate of households facing catastrophic health expenditure therefore probably underestimated the financial burden of chronic disease for households. Forth, expenditure on healthcare for chronic disease was estimated by respondents over the year prior to the survey, which may have led to some recall bias. Finally, this study is exclusively quantitative. The qualitative data which is also well acknowledged to complement the quantitative data results were not explored in the study, such as the policy implementation arrangements, policy resistance and health system bottlenecks; they may also impact the policies.

### Conclusions

Patients with chronic disease place heavy economic burden on the households associated with frequent outpatient visits and use of multiple prescription medicines. The NCMS programs in rural areas are a vital method of financial protection for the households with chronic disease. Despite a lack of statistically significant finding on the NCMS reimbursement policies on the burden of chronic illness, these programs should be strengthened both in margins of intensity (higher reimbursement rate) and extensity (e.g., other chronic disease).
